# Quetelet and the emergence of the behavioral sciences

**DOI:** 10.1186/s40064-015-1261-7

**Published:** 2015-09-04

**Authors:** Gustav Jahoda

**Affiliations:** Department of Psychology, University of Strathclyde, Glasgow, UK

**Keywords:** Quetelet forgotten, Statistics, Determinism, Influence, Nineteenth century, Behavioural sciences

## Abstract

Adolphe Quetelet was one of the most prominent figures of the second half of the nineteenth century, yet in present-day histories of several social sciences the impact of his ideas is widely ignored. The first part consists of a sketch of his life and work. Astronomer and statistician, he sought to apply the mathematical tools of astronomy to create was has been called a ‘mathematics of society’. In particular he demonstrated regularities in the incidence of various social phenomena, notably crime, whose implications were widely debated. In the second part the influence he exerted on some key figures in the then emerging social sciences is traced in some detail; these figures include Durkheim, Galton, Marx, and Tylor. He also advocated the wider use of statistics and his call had a powerful impact on the then emerging fields such as administration, economics, sociology and psychology. He influenced some of his most famous contemporaries, including Florence Nightingale, Karl Marx and Francis Galton.

Lambert Adolphe Jaques Quetelet (1796–1874) was one of the major figures of his period, justly celebrated internationally by his contemporaries. He made an enormous impact on the emerging behavioural sciences in the nineteenth century. Yet his fame is now eclipsed, as indicated by the sub-title of an article by Desrosières (1997): ‘from pedestal to oblivion’. This is not altogether true, since in general histories of the social sciences, like that by Roger Smith (1997), his ideas are usually discussed. Moreover, there is an extensive specialised literature dealing with Quetelet, much of it fairly recent. But most of it focuses on particular contributions, so that it fails to convey the whole range of his influence. In histories of various fields of social science, except for statistics and criminology where he always figures, his name rarely appears. I cannot pretend to have conducted a thorough survey, but have examined all 22 histories of psychology in the University library. All of them of course deal with Francis Galton, but none mention Quetelet. As far as psychology is concerned, it is likely that the neglect began during the inter-war years. Edwin Boring ([1929] 1950, p. 477), in his classic history of experimental psychology, showed how Galton was inspired by Quetelet when he tried to assess the distribution of mental ability (a topic treated below); and he sketched the background of Quetelet’s ideas in his Notes (p. 499). Yet Gardner Murphy ([1928] 1938), in his once very prominent history of psychology, did not refer to Quetelet at all. More recently Danziger (1990), in his important work on the origins of psychological research, calls Quetelet ‘the pioneer of statistical social science’ (p. 76) and notes his link with Galton. However, he does not elaborate on Quetelet’s contributions, nor discuss his influence on Galton.^1^

Generally, omissions were not confined to histories of psychology. Admittedly more superficial scanning of histories of anthropology and sociology, subjects to which Quetelet also made important contributions, yielded the same puzzling result.

Since familiarity with Quetelet cannot be assumed, the first section is devoted to a sketch of the man and his work. Then follows an outline of the various fields that were fertilised by his ideas, thereby demonstrating his omission to be unjustified; these fields are somewhat arbitrarily grouped into several categories. The last section provides a summary of Quetelet’s major contributions.

## On Quetelet

### Life

Quetelet’s *oeuvre* is wide-ranging and complex, and so what can be offered here is merely a greatly simplified outline of his main ideas. Born in Ghent, which later became part of Belgium, Quetelet was a man of many and diverse talents. Aged 17, he became a teacher of mathematics at a private school, and at 19 was recruited to newly established College of Ghent as an instructor in mathematics. There Quetelet was a colleague of Garnier, professor of astronomy and higher mathematics, an experience that shaped his career as a scientist. At that period he was also keen on the arts, being apprenticed to a painter and publishing poetry. In 1820 he was elected to the Royal Academy of Sciences in Brussels, and persuaded the Minister of Education that an observatory should be built. In preparation for this task he went to Paris in 1823 where he met prominent astronomers and mathematicians, including such great figures as Laplace, Fourier, and Poisson. It was they who inspired what was to become the core of his theories.

Quetelet gave lectures at the Brussels Museum on astronomy and probability, and published a popular book on astronomy. It was put on the Index of forbidden books by the Vatican, and the ensuing publicity greatly increased the size of his readership. He also conducted extensive meteorological studies, a topic then related to astronomy. In 1834 he was elected Permanent Secretary of the Brussels Academy of Arts and Sciences, and in 1836 appointed Professor of Astronomy at the newly established *Ecole Militaire* in Brussels. By that time his international fame had been well established, and continued growing until his death. Some major biographical sources are listed in a footnote.^2^

### The links between astronomy and the physiological and ‘moral’ (i.e. social) spheres

It is not known what prompted Quetelet to make this connection, which is present in his *Physique sociale* (1835). He saw an analogy between the errors of measurement in astronomical observations and those entailed in data concerned with human populations, both subject to what he called ‘the law of error’. The other key notion concerned the distribution of observations, which takes a ‘Gaussian’ form (now called a ‘normal distribution’). This he regarded as a universal principle valid in astronomy as well as in studies of humans beings (and indeed of animals and plants). In all these spheres he did consider both extremes of distributions, but saw the mean as the most probable true value. As far as humans are concerned, the difference from astronomy lay for him in the fact that data about humans are subject to multiple sources of variation, which he called ‘accidental causes’. Therefore one has to work with large numbers, where these variations cancel out; and provided the distribution is normal, which he regarded as true of most physical and social characteristics, the mean reflects a constant cause. This led him to postulate a concept that remained central to his theorising, namely that of the ‘average man’.

### *L’homme moyen* (the average man)

Originally he himself saw this as a fictitious entity, but later Quetelet treated it as a genuine *type* representative of group membership, where the group ranges from the tribe, nation or race to humanity as a whole. The specification of *l’homme moyen* in terms of various physical or social features was the objective of nearly all his researches. Moreover, he regarded *l’homme moyen* as the epitome of all qualities, physical and moral. Thus Quetelet saw this person as the most moral, the most intelligent (at any particular stage, since he believed intelligence to advance progressively over generations), the most artistic, the most beautiful, and so on. Quetelet thought that *l’homme moyen* is the product of a biological process, the kind of person nature had largely shaped. While certain aspects of this type constitute a human universal, others vary according to nationality or race and undergo changes over time. Since the type depends in part on the features of particular societies, he extended his theory to cover this aspect (cf. Halbwachs 1913). Before presenting this extension, Quetelet’s first major and most influential work (briefly mentioned above) will be outlined.

### Physique Sociale (social physics)

The term ‘social physics’ appears in the original title (Quetelet [1835] 2013) of this important volume, describing his theory and reporting his early empirical studies. Subsequent publications elaborate the main thesis without adding anything radically new. The empirical studies in this volume are mainly based on official statistics of various kinds. The first part, entitled ‘Development of the physical qualities of men’, covers statistical materials on births and mortality, ending with comparative population statistics and their implications. The second part traces the development with age of such physical features as height and weight, considered as manifestations of general laws. For instance, a table is headed: ‘Law of the growth of women’ (Quetelet [1835] 2013, p. 63).

The third part, which attracted most attention and set off heated controversies, concerns the ‘moral and intellectual qualities of man’. It was a daring and entirely novel attempt to subject to statistical analysis the kinds of topics until then usually reserved for philosophical discussions. Quetelet lacked the tools to deal systematically with intelligence, and so he fell back on records of achievement. In principle that was not a bad idea; but since he confined it to comparisons of French and English dramatists, it was not really illuminating. More interesting are tables of the incidence of mental illness across several nations. However, the area which commentators highlighted was that of ‘moral qualities’. Among these the topic of crime figures most prominently, no doubt since it was one where ample data were available. That made it possible to undertake numerous sophisticated analyses, including even curve-fitting. It is not possible here to go into details, and only the sensational conclusion, adumbrated at the beginning of the book, will be quoted:

The remarkable constancy with which the same crimes appear annually in the same order, drawing down on their perpetrators the same punishments, in the same proportions, is a singular fact… We might even predict annually how many individuals will stain their hands with the blood of their fellow-men, how many will be forgers, how many will deal in poison, pretty nearly in the same way as we may foretell the annual births and deaths. (Quetelet [1835] 2013, pp. 6–7)

This regularity, which at first astonished Quetelet himself, was attributed by him to the nature of society, to which he subsequently devoted another work.

### On the social system and the laws that govern it (Quetelet 1848)

When Quetelet mentions ‘laws’, as he does in the title of this book, it is not always clear whether he meant the term in a strict sense, such the laws of physics, or whether he referred merely to context-bound regularities. In the case of the social system it was certainly the latter. The task he set himself was that of describing the functioning of what he also called the ‘social body’, thereby hinting at an analogy with the individual one. The analogy is also shown by his discussing what he called the physiology of the ‘social body’, which can have illnesses and is liable to change within certain limits. For Quetelet there existed different kinds of social bodies in a hierarchy, humanity being the ultimate one where free will is entirely eliminated and the laws are as firm as those which explain the movements of heavenly bodies; but most of time he is thinking of the nation or ‘society’ which is subject to change over time; and therefore so are its laws.

According to Quetelet there are constant and variable forces operative in society. The former are geographical features which favour or hinder the level of civilization as well as causing the emergence of different races. The variable ones are traditions and customs as well as capricious collective opinions. In well-organised societies these forces cancel each other out and there prevails an equilibrium, albeit a gradually moving one since humans are capable of modifying their conditions of life. Thereby they create ‘perturbations’ which affect the equilibrium, but only within narrow limits. As regards the collective level, he expressed his conviction about the operation of fixed laws in his *Letters on probability* (Quetelet [1845] 1849, p. 178):

[The social body] subsists in virtue of conservative principles, as does everything which has proceeded from the hands of the Almighty: it also has its Physiology… we find laws as fixed as those which govern the heavenly bodies: we return to the phenomena of physics, where the freewill of man is entirely effaced, so that the work of the Creator may predominate without hindrance. The collection of these laws, which exist independently of time and of the caprices of man, form a separate science, which I have considered myself entitled to name social physics.

In spite this insistence on lawfulness, Quetelet emphasized that this did not imply stagnation—like most of his contemporaries he believed in progress. In fact he thought that his teachings could contribute to it by encouraging the use of statistics in various fields of knowledge.

### Example of application of statistics

In the course of their education Albert (future Consort to Queen Victoria) and his brother spent some time in Brussels, where Quetelet was one of their tutors. In the above-mentioned *Letters on probability* sent to Albert, he dealt not merely with theory but also discussed several potential practical applications of statistics related to such spheres as administration.

As will appear in due course, one of these chapters is specially relevant in the present context. Entitled ‘The use of statistics in the medical sciences’, its aim was to counter the prevalent scepticism of medical men regarding statistics. It may be mentioned in passing that attitudes about which Quetelet complained were not confined to his period. Over a century later Richard Asher (1972), author of a critical medical book noted a dislike of statistics, particularly on the part of fashionable and successful doctors. At any rate, on the basis of statistical considerations, Quetelet pointed out a series of weaknesses in medical practice. One is the attribution by doctors of different causes and the use of different remedies with regard to the same illness. This he said was due to inadequate record keeping, coupled with faulty memory and inadequate communication. Consequently no real connection is established between the supposed cause and the remedy, and the difficulty of doing so is increased by the fact that there might be multiple causes. Quetelet suggested what has now become well understood, namely the need to compare the effectiveness of medicines ‘… by inquiring what would become of [an ill person] if abandoned to the force of nature only’. The problem is less acute when it comes to public health, where calculation of probabilities is helpful in establishing causal factors. As far as hospitals are concerned, statistics collected from the major ones in Europe show that the mortality rates varies more with their directors than with their therapies: ‘I would say that a good administration perhaps saves more patients than the science of the most skilful doctors.’ (Quetelet [1845] 1849, pp. 235–6).

As the above shows, Quetelet was not just a theorist but had some shrewd notions about how statistics could be usefully applied. This came to be recognised in due course by many of his contemporaries; and some of the ways in which his ideas resonated during the second half of the nineteenth century among prominent figures will now be discussed.

## Illustrations of Quetelet’s influence in various fields

### History and philosophy

The Newtonian revolution made a huge impact on European thought. Several notable thinkers, inspired by Newton’s demonstration of the lawfulness of the physical universe, began to ask the question whether there might not be analogous causal laws governing mind and society. Among those who raised such issues was Condorcet (1743–1794) who believed that the ‘moral sciences’ were basically not inferior to the physical ones. An even more radically determinist view was held by Laplace (1749–1827), who famously declared that his ‘demon’, a super-intellect, could in principle forecast the future of everything in the universe. Although towards the end of the eighteenth century a reaction set in against the rationalist spirit of the Enlightenment, a strong vein of the earlier trend persisted. It took several similar forms and came under such labels as ‘determinism’, ‘materialism’ or (Comtian) ‘positivism’. While distinct notions, common trends among them included a rejection of romanticism with its emphasis on emotion and free will. In the clash between these contrasting ideologies, history and philosophy cannot be readily distinguished.

A key figure who sparked off debates that dominated the second half of the nineteenth century was Henry Thomas Buckle (1821–1862), an inheritor of the spirit of the Enlightenment.^3^ Buckle was a remarkable man. Self-educated, he learned no fewer than eighteen languages and was a brilliant chess player. In 1857 he began to publish a monumental *History of civilization in England* (Buckle [1857] 1904) that was never completed. Yet what he did publish had an immense impact on his contemporaries, not only on scholars but also on the educated public; and not just in Britain: there were translations into French, German, Russian, and other languages.

In the beginning of that work Buckle discusses Quetelet at considerable length and uses him to underpin his thesis that history is subject to deterministic laws, and therefore ought to become a science in the same manner as the natural sciences. Buckle cites Quetelet on the regularities to be found in social life; for example he rehearses Qetelet’s finding that the number of murders per year remains much the same, and that this even applies to the proportions of different killing methods employed. The conclusions Buckle draws form this are spelled out in a famous passage about suicide. After describing ‘self-murder’ as seemingly ‘capricious and uncontrollable’, he goes on as follows:

… it is surely an astonishing fact that all the evidence we possess respecting [suicide] points to one great conclusion, and can leave no doubt on our minds that suicide is merely the product of the general condition of society, and that the individual felon only carries into effect what is a necessary consequence of preceding circumstances. In a given state of society a certain number of persons must put an end to their own life. This is the general law; and the special question as to who shall commit the crime depends of course upon special laws; which, however in their total action must obey the large social law to which they are all subordinate. And the power of the larger law is so irresistible, that neither the love of life nor the fear of another world can avail anything towards even checking its operation. (Buckle [1857] 1904, pp. 15–16).

It was the extraordinary success of Buckle’s *History* that brought Quetelet to the notice of an international readership, and thereby helped to spread his fame.

Buckle, believing himself to have established the existence of deterministic historical laws on the basis of Quetelet’s theory, proceeded for the remainder of his *magnum opus* to deploy an exceedingly ambitious historical narrative—yet devoid of any statistics. Buckle attacked his fellow-historians for their narrow specialization and unwillingness or inability to generalise. He himself did not hesitate to do so, and some of the views he expressed, (e.g. about relationship between climate, food, population and the distribution of wealth) are plausible. Others, like his notion that intellectual development is influenced by the extent to which Nature is violent (such as earthquakes), seem bizarre. At any rate he made no claim to having discovered historical laws analogous to those governing celestial motions—that he regarded as a task for the future.

Buckle’s radical claims had significant repercussions. Almost at once there were critics, and as far as Buckle’s historical analysis was concerned, they were largely successful in demolishing it. However, his contention that history could and should become a science set off intense debates. Almost every prominent figure of the period took some part in them. The story of how historians reacted is described in detail by Ian Hesketh (2011). Philosophers like John Stuart Mill discussed the issues, and even distinguished scientists like Darwin and Clerk Maxwell commented on Buckle’s views about the nature of scientific method.

Buckle’s remarkable success also had wider implications for philosophical issues. While Quetelet regarded only large-scale social phenomena as subject to law, Buckle went well beyond that in holding that *all* individual human actions are also lawfully determined. This of course amounted to a denial of the possibility of free will, thereby undermining basic tenets of Christianity. The ensuing debate was the first of its kind only in so far as it turned on the implications of statistical regularities. In Britain a major figure writing in defence of free will was the philosopher of probability, John Venn (1866); he rejected the notion that social events can occur independently of human motives, a doctrine he called ‘fatalism’. In Germany the debate was more wide-ranging, probably because materialism was more widespread there at the time. 4

### Administration and economics

Quetelet’s direct influence on administration was most pronounced in the case of Florence Nightingale (1820–1910). Known to the public as the ‘Lady of the Lamp’, her popular fame is based on her work with injured and diseased soldiers during the Crimean War. However, her achievements were far more extensive. When she returned from the Crimea to Britain in 1856, she embarked on a mission to bring about sanitary reforms and gained the support of many prominent figures. As regards statistics, she obtained the help of William Farr, a medical doctor who had become an expert on statistics of public health, and they collaborated over many years.

It is not clear when Florence Nightingale first came across Quetelet’s writings. Edward T. Cook (1913), her biographer claims that she had read the first edition of the *Physique Sociale* (1835), but that is doubtful. What is certain is that she was familiar with Quetelet’s ([1845] 1849) *Letters on probability theory*, since she said that ‘Quetelet’s chapter on medicine alone is a book for a whole Profession to work out’.^5^ This chapter is briefly outlined above, at the end of the section on Quetelet’s life.

The only personal meeting between Florence Nightingale and Quetelet took place in 1860 at the International Statistical Congress held in London. By that time she had become an invalid and Quetelet as well as other delegates visited her at home. They remained in contact by correspondence, and he presented her with the two volumes of the 1869 edition of the *Physique Sociale*. Florence Nightingale’s response, which shows the extent of her admiration, began as follows: ‘Homage to Monsieur Quetelet, creator of statistics…’.^6^ Pearson (1914–1930, vol. II, p. 414) who saw the original copies, says that every page was annotated by her. She was rightly dubbed a ‘passionate statistician’, since her devotion to this field was imbued with religious fervour (McDonald 1998). She felt that conducting social research was a way of discovering divine laws designed to bring about moral progress. Here again her thinking was in harmony with that of Quetelet, as is clear from the passage from his *Letters* cited above, where he referred to ‘the hands of the Almighty’. Florence Nightingale’s faith was in complete harmony with the spirit of Quetelet, and she seems to have been ready to accept almost any of his pronouncements.^7^

In sum, it was Florence Nightingale’s conviction that the use of statistics is essential not just for administrators, but also for legislators and politicians whose plans should be based on the relevant knowledge—otherwise they risk failure. She even attempted, together with Francis Galton, to establish university courses of statistics; but that was not successful at the time.

While Florence Nightingale was concerned with the practical applications of statistics, the writings of William Stanley Jevons (1835–1882) dealt with economic theory. He was thoroughly familiar with Quetelet’s work and made extensive use of his ideas (Mosselmans 2005; White 1994). Jevons quoted extensively from the *Letters on Probability*, including the ‘law of error’ and particularly the concept of the ‘average man’. Jevons transposed that concept to the body of consumers and their behaviours. The rate of consumption of the collectivity was said to be governed by price, while the purchasing behaviour of individuals depends on their individual motives and resources and will therefore differ widely. However, like Quetelet, Jevons suggests that in the aggregate these particular ‘accidental’ factors will cancel each other out. The theory is of course much further elaborated, but its main thesis owes a great deal to Quetelet.

A much more important figure In economics and politics was Karl Marx (1818–1883). Although less is known in detail about his debt to Quetelet, there is no doubt that he was well acquainted with his work (cf. Wells 2013) Marx seldom quoted him, but there are exceptions. In an article on capital punishment in the *New York Tribune* (February 18, 1853) he writes ‘Mr. A. Quetelet, in this excellent and learned work l’Homme et ses facultés…’.

There are only two passages in *Capital*, where he refers to Quetelet: in connection with the issue of surplus value Marx discusses problems of individual differences and the elimination of ‘random errors’ by averaging (Marx [1867] 1981, vol. 1, p. 440); and he agrees with Quetelet on the nature of statistical regularities when he states that ‘the law of value’ is visible only when accidental variations are combined in large numbers’ (Marx [1867] 1981, vol. 3, p. 976); Furthermore, the whole structure of his approach in terms of lawfulness is reminiscent of Quetelet’s arguments in *On the social system and the laws that govern it.* This issue of lawfulness is discussed by Enfield (1976). It is hardly necessary to point out the far-reaching consequences of the ideology to whose formation Quetelet contributed.

### Sociology and anthropology

The word ‘sociology’ is doubly connected to Quetelet. Auguste Comte (1798–1857) had initially called his approach to the study of society ‘social physics’, in spite of the fact that his ‘positive philosophy’ was devoid of quantitative elements. Yet when Quetelet adopted that term, Comte abandoned it for fear of being regarded merely as a follower; instead, he invented the term ‘sociology’. The other connection is that Quetelet saw himself as founding the scientific study of society by means of statistics, and as such creating a kind of sociology. It is therefore not surprising that one of the most prominent sociologists of the late nineteenth century was influenced by him.

Emile Durkheim (1858–1917) first took up some of Quetelet’s ideas and modified them in his book on *The rules of sociological method* (Durkheim [1895] 1982). There he treated Quetelet’s ‘average’ type as representing the normal as distinct from the deviant; moreover, he linked it to the collective mind, which he regarded as the determinant of the regularities in social phenomena such as the crime rate. This line of thought was further developed in the volume on suicide, which was one of the first sociological studies following Quetelet’s lead by using statistics.

In his classic work on suicide (Durkheim [1897] 1952) he starts by outlining Quetelet’s main thesis and then proceeds to an elaborate critique—largely justified it must be said. His main critique concerns the inferences based on the concept of the ‘average man’. It will be recalled that Quetelet had sought to explain the regularity of crimes and suicides on the basis that the ‘average man’ has some disposition towards crime and suicide, but only those who deviate strongly enough actually commit these acts. Now as regards suicide, Durkheim points out that it is much rarer than crime, so that the probability that any individual will commit suicide is close to zero. More generally, he criticises Quetelet’s focus on the individual which, given the variability of dispositions such as motivations, cannot serve as an explanation. Durkheim himself maintained that that there are external forces independent from and impinging on the individual from the outside, namely ‘collective representations’. It is these, he argues, which account for the regularities in the rates of suicide within particular nations. It is a thesis that has been widely debated among sociologists.

In view of Quetelet’s aim to create what has been called a ‘mathematics of society’ (Porter 1985), it is paradoxical that sociology is the one sphere where Quetelet’s ideas have left little trace. There was a long gap between Durkheim’s use of statistics and the wider adoption of statistical methods in more recent sociology, where Quetelet’s name is seldom if ever mentioned.

Another contemporary of Quetelet was Edward Burnett Tylor (1832–1917), sometimes known as ‘the father of anthropology’. While not the first to use the word ‘culture’ more or less in its modern sense, he can certainly be credited with defining the concept and ensuring its wide adoption. One of his major works is entitled *Primitive Culture* (Tylor 1871), and in its introductory pages one reads: ‘To many educated minds there seems to be something presumptuous and repulsive in the view that the history of mankind is part and parcel of the history of nature, that our thoughts, wills, and actions accord with laws as definite as those which govern the motions of the waves, the combinations of acids and bases, and the growth if plants and animals’ (Tylor [1871] 1958, p. 2). A few pages later he refers directly to Quetelet and details the regularity of phenomena such as crime rates, exactly as documented by Quetelet. The following year he published a review of two of Quetelet’s major works, namely *Physique Sociale* and *Anthropométrie* (Tylor 1872). In this piece he refers with approval to Thomas Buckle’s doctrine of history’s inexorable laws. He also tries to deal with the objection that humans have free will, by saying that free will is of limited range—sufficient to prevent us from predicting the behaviour of individuals, but ceasing when it comes to the collective actions of large numbers of people.

In this connection Tylor discusses Quetelet’s concept of ‘the average man’, explaining that it refers to the modal type around which the population of a nation or race clusters, and which is a kind of representation of the whole. He mentions the measurements of the heights of 26,000 American soldiers, used to show that the distributions conforms to the ‘law of binomial expansion’. This approach, he contends, will make it possible to clearly characterise a nation or a race as contrasted with the usual vague ideas. Tylor further notes that in the *Anthropométrie* ‘M. Quetelet finds a small number of selected individuals sufficient for ascertaining the standard national proportions..’ (Tylor 1872, p. 363). Now given that Quetelet usually dealt with large numbers, that calls for some elucidation.

Near the beginning of Quetelet’s *Anthropométrie* (1870, pp. 23–24), one is surprised to find the following passage:

Are the accidental causes which divide people within a given country sufficiently numerous and influential that it is necessary to resort to a large number of persons in order to eliminate the particular features they present? Experiment teaches us that the answer is negative. [There follows a brief account of the ‘experiments without sufficient details; Quetelet then concludes that]… it has in effect become unnecessary to have recourse to a great number of persons, since ten were generally sufficient to provide a mean value that departed little from the type I had intended to determine. [my translation]

This procedure certainly did not entail random sampling, as is clear from the parts of the volume dealing with different human races. Taking advantage of the presence in Brussels of individuals belonging to other races, he measured them in his usual exceedingly detailed manner. So one finds tables comparing three young American Indians (O-jib-be-wass), ten Belgian soldiers and, curiously, a white American athlete; or again, even more absurdly, one Chinese man and one Chinese woman are compared with one Belgian of each sex. A reasonable explanation for such aberrations, offered by Quetelet’s biographer Lottin, is that, after a stroke in 1855, his intellectual powers suffered a sharp decline.^8^

It is now possible to understand Tylor’s contention that small numbers of ‘typical’ individuals are sufficient to arrive at the key features of an ethnic group. He recommends this method to anthropologists, and in a footnote he offers as an example a brief report on the heights of thirty Chippewa Indians by General Lefroy (1870). On consulting this report one finds that these Indians unexpectedly turned up at a trading station and were dragooned into being measured. Whether or not they were ‘typical’ is an open question. Later generations of [especially British] anthropologists did in fact usually rely on a small number of ‘informants’. However, that was probably in the belief that they were tapping Durkheim’s ‘collective representations’ rather than owing to Tylor having followed Quetelet’s erroneous suggestion.

A more significant contribution by Tylor, inspired partly by Quetelet and partly by Adolphe Bastian (who had stressed the importance of statistics for anthropology) was his pioneering study of ‘adhesions’ between social institutions (Tylor 1889). In this he tried to establish the social-evolutionary development of institutions by tracing what we would call ‘correlations’ between them.^9^ This led ultimately to the setting up of the Human Relations Area Files (HRAF) at Yale, which is still used test hypotheses about cultural elements across the globe.

### Psychology

The bulk of Quetelet’s empirical work dealt with physical features of humans and crime rates, but he was also interested in what he called man’s intellectual qualities. However, as noted above, his approach was not very fruitful. The problem was taken up by Francis Galton (1822–1911), cousin of Darwin, who was a many-sided genius interested in psychometry and statistics. Galton was inspired by Quetelet, though he tackled the issue of intelligence in quite a different manner. In the preface to *Hereditary genius* ([1869] 1914, p. 11) he describes how the ‘law of error’ had been applied by Quetelet

… to the proportions of the human body, on the grounds that the differences, say in stature, between men of the same race might *theoretically* be treated as if they were Errors [sic] made by Nature in her attempt to mould individual men… according to the same ideal pattern [i.e. the average man]. Fantastic as such a notion may appear… it can be shown to rest on a perfectly just basis.

This conception, he notes, had enabled Quetelet to make theoretical predictions about body dimensions that fitted the observed data. Galton then took a crucial jump:

Now, if this be the case with stature, then it will be true as regards every other physical feature – as circumference of head, size of brain, weight of grey matter, number of brain fibres, etc.; and thence, by a step on which no physiologist will hesitate, as regards mental capacity. (Galton [1869] 1914, p. 28)

Unlike Quetelet, who had focused on the average, Galton considered the whole of the normal curve and took a radical step by postulating that it reflected the distribution of mental ability in a population—an assumption that underlies all subsequent intelligence-testing. He tried out this hunch in relation to examination marks, making theoretical predictions on the basis of the normal curve and demonstrating a good fit between predictions and actual results. Thereby encouraged, Galton devised a set of seven—admittedly arbitrary—grades of mental ability from A to G, assuming the intervals between them to be equal. It is not necessary here to go into the details of his rather complex scheme, but some of its salient features will be outlined. Using census data for England and Wales as his basis, he calculated the frequency of each grade per million individuals. It should also be explained that, unusually for us, he divided each grade into two parts above and below the median, the former having greater and the latter lesser ability. The first grade clusters about the median and comprises one in every four people. Together with the second grade, this accounts for the bulk of the population which he characterised as ‘mediocre’. In order to make this more concrete, he described it as typical of people in provincial gatherings. The upper half of Grade C, he suggested, corresponds roughly to the ability of the foreman of an ordinary jury. At the extreme ends of the distribution, namely the two parts of G, are the extraordinarily rare geniuses at the top and idiots at the bottom. At the end of this section he comments on the huge range of ability among humans.

As mentioned above, Quetelet had also discussed the characteristics human races, and this may well have led Galton to address that theme, as he did towards the end of the volume. However, he again tackled this in a different and more systematic way. Referring to Darwin’s law of natural selection, Galton argues that the evolutionary process will have differentially shaped the abilities of different races. In evaluating comparative abilities, he claims to have made use of ‘the law of deviation from the average’, though no numerical data were available. In arriving at what he modestly calls ‘a rough provisional inquiry’, he once again assumed that the intervals are the same in all races so that ‘If the ability of class A in one race be equal to the ability of class C in another, then the ability of class B of the former shall be supposed equal to that of class D of the latter, and so on.’ (Galton [1869] 1914, p. 326).

The only ethnic group dealt with in some detail was the ‘Negro race’, which he compared with the Anglo-Saxon one. He begins by acknowledging that the ‘negro [sic] race’ in America is handicapped by social disabilities and therefore they cannot be fairly compared—hence the need ‘for much rougher data’. For that purpose he looked at some ‘occasional’ high achievers such as Toussaint l’Ouverture, whom he regarded as atypical. This led him to suggest that blacks are at least two grades below those of whites. Then he admitted that there are blacks clearly superior to the average of whites, which would mean that they were of class C or even D.

In another line of argument Galton considers Africans in their own country, conceding that ‘a native chief is well versed in the art of ruling men’; nonetheless he contends that a visiting European traveller never has any trouble dealing with them. Finally, Galton claims that a very large number of Negroes are ‘half-witted’: ‘Every book alluding to negro servants in America is full of instances. I was myself much impressed by this fact during my travels in Africa. The mistakes the negroes made in their own matters were so childish, stupid, and simpleton-like, as frequently to make me ashamed of my own species’ (Galton [1869] 1914, p. 328). The notion of the stupidity of black servants persisted well into the twentieth century. A biologist as distinguished as Julian Huxley made the same observation in his younger days (Huxley 1924).^10^ The remainder of the chapter consists of somewhat diffuse remarks. Thus Galton reckoned that ‘the Australian type’ is probably at least one grade below Africans, that Lowland Scots and people from the north of England are definitely ‘a fraction of a grade’ above the ‘ordinary English’, and he ends by speculating about the ancient Greeks, as Quetelet had done.

The grading of mental abilities was not the only area in which Galton built upon the foundations laid by Quetelet. Another was that of composite photographs (Galton 1879), where pictures of particular categories of people are superimposed. They are said to represent various *types* such as criminals (a line later famously followed by Cesare Lombroso ([1876] 1911). In that paper Galton explains its title (Generic images) as follows: ‘The word generic presupposes a genus, that is to say a collection of individuals who have much in common, and among whom medium characteristics are very much more frequent than extreme ones. The same idea is sometimes expressed by the word typical, which was much used by Quetelet, who was the first to give it a rigorous interpretation, and whose idea of a type lies at the basis of his statistical views’ (Galton 1879, p. 162). He further applied this notion to the concept of ‘race’ saying that it constitutes a typical form around which various features cluster, and that will also be true of their descendants. Galton also directly compared composite photographs to Quetelet’s ‘homme moyen’. All this shows Galton’s considerable indebtedness to Quetelet.

The above survey, designed to illustrate the pervasive influence of Quetelet’s ideas throughout the second half of the nineteenth century, is of course far from comprehensive. His contribution to the development of statistical methods, while certainly not negligible, is probably less significant than the efforts he made over the years to encourage in various countries the systematic collection of standardised statistical information. This he envisaged in such fields as population, economics, medicine, and what he called ‘morals’ (of which crime was the most salient). Quetelet was further instrumental in organising the first international congress of statistics in 1853, intended to bring together people working in offices of statistics, for whose creation he had often been responsible. In sum, he played a major role in initiating the collection of statistics, information essential for the functioning of modern society (cf. Derosières 2002). Finally, there is one concept he elaborated that is still extensively applied at present, namely BMI, the ‘Body Mass Index’ (Eknoyan 2008).

It should be clear from the above that Quetelet’s ideas were among the main driving forces generating the behavioural sciences as we know them today. In his own time his brilliance was generally recognized and he enjoyed the esteem that was his due. His ideas, as has been shown, were borrowed by others and incorporated into their own opus, often with little or no acknowledgement of their source as a few examples will show. The immense intellectual edifice constructed by Marx incorporates some of Quetelet’s basic tenets, as is revealed by the key passage below:

Also when a society has discovered the *natural laws* of its development – and it the ultimate purpose of this work to unveil the *law* of the economic development of modern society – it can neither leapfrog the *natural* phases of development, nor make them go away by issuing a decree. (my emphases and translation) (Marx 1867, p. 14)

This phrasing, with its stress on the natural character of societal changes, closely echoes Quetelet’s formulations; yet Marx merely mentions him in a footnote.

Tylor’s influential piece on ‘adhesions’, which ultimately resulted in the HRAF, employs Quetelet’s language (e.g. ‘laws of error’) but his name does not appear. Again, Quetelet’s project was that of pioneering a ‘social physics’, or as we would say a quantitative sociology. Yet John Stuart Mill (1879, II, p. 490) wrote about Comte that he was ‘the only thinker who, with a competent knowledge of scientific methods in general, has attempted to characterize the Method of Sociology…’; and that in spite of the fact that Comte’s system lacks any quantitative element. So the admirers of Comte ensured that he came to be regarded as the founder of sociology.

Enough has probably been said to show how others made use of Quetelet’s ideas in their own work which ensured their fame, a fame that survives to the present. By contrast, Quetelet himself has sunk into obscurity within several of the disciplines he had helped to create: that is surely an injustice, and he deserves to be remembered.

## Footnotes

1 Danziger’s (1990) book is a landmark because he demonstrates historically how and when statistical thinking came to predominate in experimental psychology.

2 Edward Mailly (1875) has a long and detailed obituary. The main biography is by Joseph Lottin (1912), which has been reprinted in 2012 (Memphis, USA: General Books); however, that text is full of errors and omissions. Useful biographical details can be found in the following: Frank Hankins ([1908] 1986); Theodor M. Porter (1986), and Oscar Shynin (1986).

3 For details of his life see Giles St. Aubyn (1958).

4 Buckle’s thesis was widely discussed on the continent, notably in Germany. For instance, on the issue of history as a science see Johann Droysden (1863). As regards determinism versus free will, Adolph Wagner (1864) tends to support the former, while Moritz Drobisch (1867) writing in the Kantian tradition proposes that the stability of the will accounts for the regularity of social statistics.

5 Cited in Diamond and Stone (1981, p. 73).

6 Cited in Diamond and Stone (1981, p. 71).

7 This is shown by a story recorded by her biographer, which also illustrates the wide range of Quetelet’s interests. Based on his work in meteorology, he proposed a law governing the flowering of plants. It is said to occur when ‘…the sum of the squares of the mean daily temperature, counted from the end of the frosts, equals 4246 degrees C.’ Apparently Florence Nightingale suggested to her gardening friends that they should do these calculations in order to verify the law. It is not stated whether any of them followed this suggestion (Cook 1913, vol. 1, pp. 429–30).

8 Quetelet’s lack of concern with sampling issues and his tendency to make wide generalizations from a few cases persists even today. It happens especially in experimental social psychology, where a small number of students are sometimes taken to represent humanity at large.

9 Galton, who was present when Tylor expounded his thesis, pointed out a possible source of error which is still known as ‘Galton’s problem’.

10 In fairness it should be mentioned that Huxley later became a strong opponent of Racism.
